# Editorial: Biomarker discovery and validation in neurological diseases

**DOI:** 10.3389/fncel.2026.1877174

**Published:** 2026-05-27

**Authors:** Shivani Singla, Bhupesh Vaidya, Laura Martínez-Palma, Dirk M. Hermann

**Affiliations:** 1 Texas Medical Center, Houston, TX, United States; 2 Academic Unit Histology & Embryology, Facultad de Medicina, Universidad de la República, Montevideo, Uruguay; 3 ALS Center of Uruguay (CELAU), Facultad de Medicina, Universidad de la República, Montevideo, Uruguay; 4 Department of Neurology, University Hospital Essen, University of Duisburg-Essen, Essen, Germany

**Keywords:** ischemic stroke, multiple sclerosis, neurosyphilis, polyneuropathy, primary central nervous system lymphoma, subarachnoid hemorrhage, tau, trigeminal neuralgia

Neurological diseases affect approximately one-third of the world's population across their life-time (GBD 2021 Nervous System Disorders Collaborators, 2024). Despite such a high prevalence, there is limited understanding of the pathologies and molecular pathways involved in these diseases. Besides, a lot of times, the symptoms of these diseases tend to overlap, which results in inaccurate diagnosis and subsequent inaccurate drug prescriptions. Though recent developments in omics technologies have accelerated drug discovery and diagnostics, there remains a dearth of accurate biomarkers capable of reliably diagnosing and predicting the clinical course of neurological diseases.

This Research Topic aims to address gaps in the literature and compile a collection of original research articles, reviews, and case reports focused on identifying and validating biomarkers for neurological disorders. The studies encompass a range of methodologies, including omics, clinical assessments, and meta-analysis, offering a multifaceted perspective on biomarker discovery in stroke, subarachnoid hemorrhage, central nervous system lymphomas, trigeminal neuralgia, neurosyphilis, multiple sclerosis, and other neurological disorders.

A central theme emerging from the Research Topic suggests the potential of using routine clinical parameters as a predictive biomarker for neurological disorders. For example, Wu et al. developed a clinical nomogram that uses serum calcium dynamics to predict delayed hydrocephalus after spontaneous subarachnoid hemorrhage. Similarly, Dong et al. reported that the lactate dehydrogenase-to-albumin ratio is a predictor of acute kidney injury in patients with intracerebral hemorrhage, potentially helping to stratify high-risk patients for improved clinical outcomes. Yu et al. further extended this framework by accounting for economic factors and routine clinical examinations when predicting stroke-associated infection in patients with acute ischemic stroke patients treated with thrombolysis. In addition, routine hematological profiling of alkaline phosphatase, creatinine, and other liver function enzymes was found to provide important reference for clinicians to monitor the health status of children with spastic cerebral palsy (Mo et al.). Together, these studies exhibit high translational relevance and could be easily integrated into routine

diagnosis without requiring sophisticated instrumentation for detailed analysis. However, it is also important to consider cross-laboratory validations across multiple centers before generalizing these findings across different epidemiological regions and hospital settings.

Another important highlight of the Research Topic is the emerging role of blood- and cerebrospinal fluid (CSF)-based biomarkers, which could provide more specific information on neurological disorders beyond that obtained through routine diagnostics. Such biomarkers have attracted increasing clinical and research interest in recent years. A study by Zhu et al. found that patients with high CSF exosomal ANGPTL2 levels had shorter progression-free survival in primary central nervous system lymphoma, which could have important clinical relevance soon. Additionally, the diagnostic and prognostic value of inflammatory markers and chemokines, including CXCL13, CXCL10, and CXCL8, was demonstrated in patients with neurosyphilis by Guan et al., underscoring the growing role of inflammation in neurological disorders. Chen et al. present a case report that correlated CSF bilirubin levels with imminent risk of bilirubin neurotoxicity in preterm infants, particularly when serum bilirubin levels are misleadingly low.

The Research Topic also highlights the growing importance of omics and systems-level approaches in biomarker discovery and validation. A systematic review by Grigolashvili et al. identified circulating microRNAs across etiopathogenetic subtypes of cerebral ischemia, highlighting the potential of miRNA-based biomarkers for disease stratification. Furthermore, the growing importance of metabolomic profiling and its use for pediatric demyelinating disorders was outlined by De Gaspari et al. as a part of this Research Topic.

The present topic also includes a case report that highlights the importance of diagnostic heterogeneity and clinical context in identifying neurological disorders. The case of POEMS syndrome (a rare multisystem paraneoplastic disorder) reported by Xing et al. as misdiagnosed as liver cirrhosis, emphasizes this issue, which can have life-threatening long-term systemic implications for the patients.

Finally, Zhang et al. emphasize that, although it is very important to have biomarkers for neurological disorders, the contribution of demographics should not be ignored in drawing accurate clinical conclusions, especially in diseases like trigeminal neuralgia. Moreover, a systematic review and meta-analysis by Hou et al. revealed reduced tau protein levels in patients with schizophrenia, which could expand the relevance of neurodegeneration-associated biomarkers into the field of psychiatric disorders.

Looking ahead, the future of biomarker discovery in neurological diseases holds great promise for patient management, and encompassing a battery of tests, including routine clinical variables, biofluid markers, omics technologies, and computational modeling, could help in improved diagnosis, improved patient stratification, better prediction of complications, and improved therapeutic decision-making.
